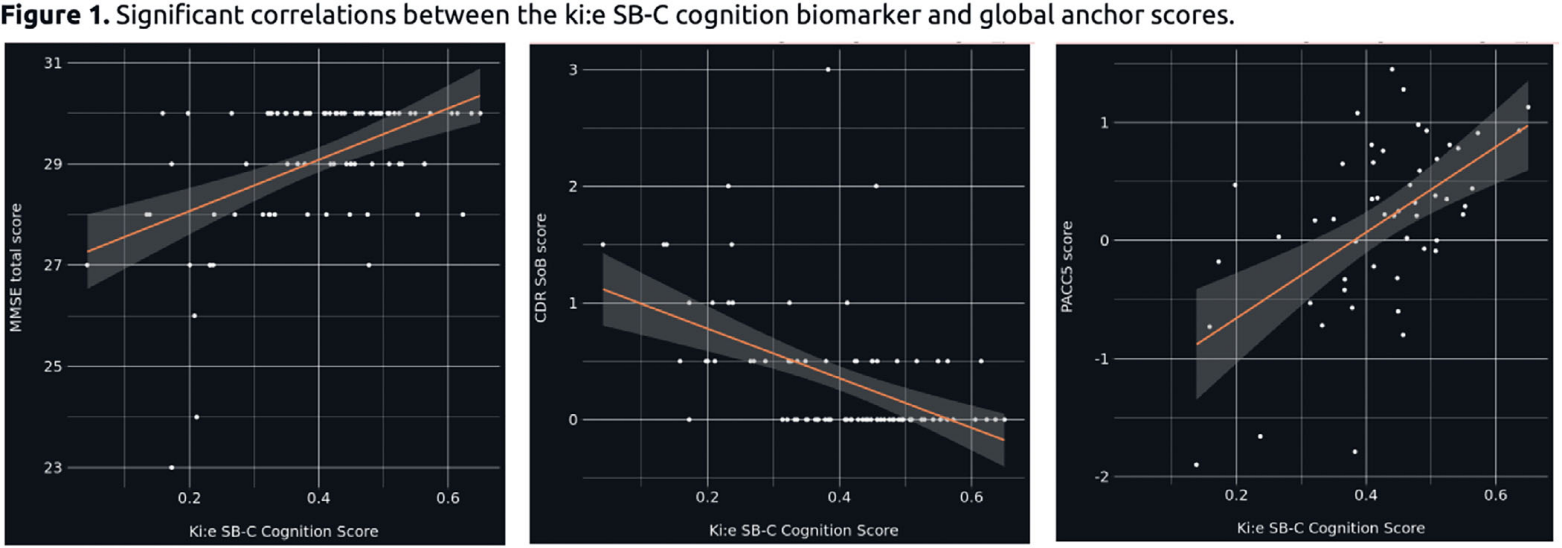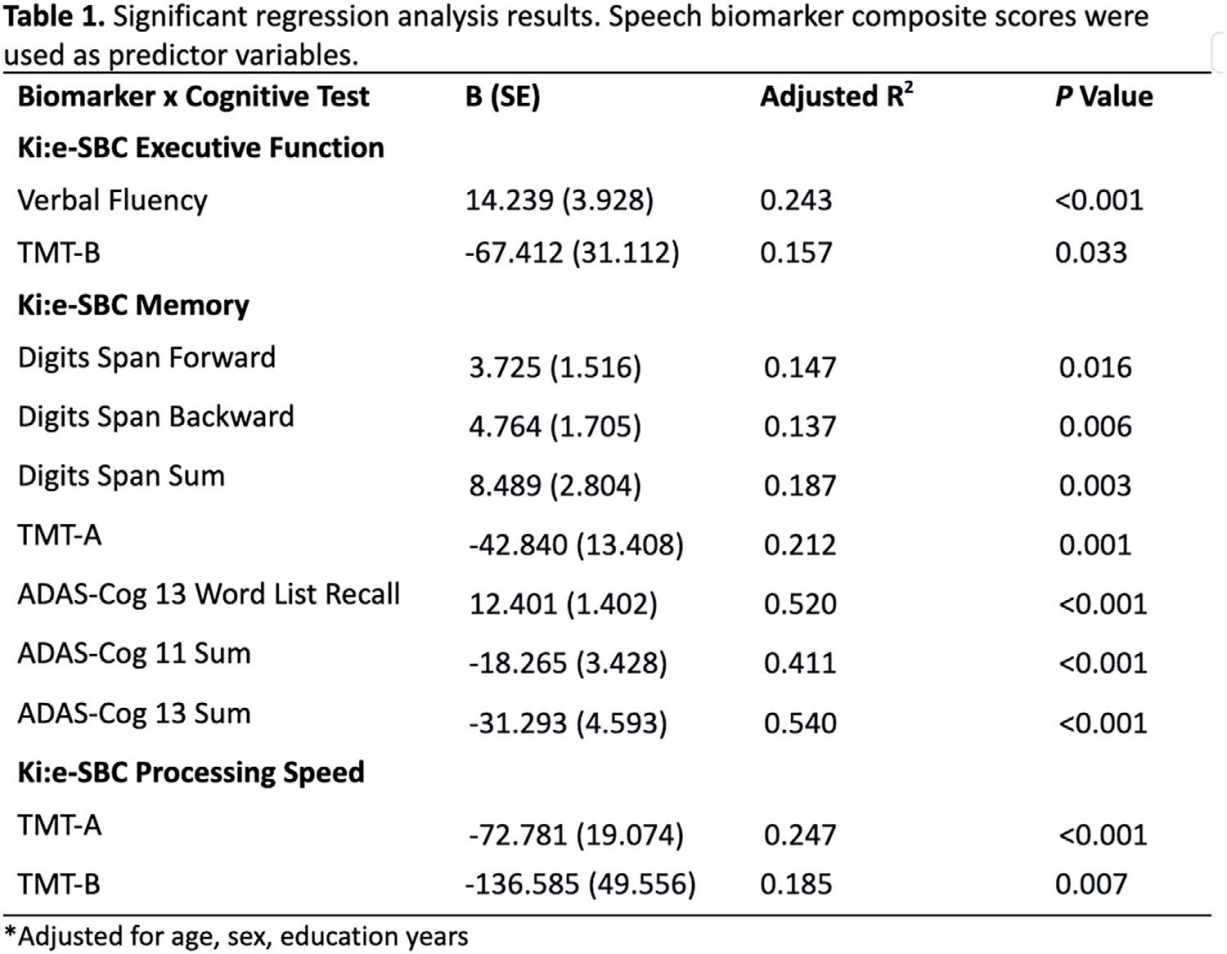# Associations Between Speech‐based Biomarkers of Cognition and Traditional Neuropsychological Assessments in a Preclinical AD Cohort

**DOI:** 10.1002/alz.084094

**Published:** 2025-01-03

**Authors:** Zampeta‐Sofia Alexopoulou, Stefanie Köhler, Johannes Tröger, Elisa Mallick, Nicklas Linz, Josef Priller, Anja Schneider, Klaus Fließbach, Björn Falkenburger, Jens Wiltfang, Inga Zerr, Frank Jessen, Thomas Klockgether, Emrah Düzel, Annika Spottke, Michael Wagner, Christoph Laske, Valeria Manera, Stefan Teipel, Alexandra König

**Affiliations:** ^1^ CoBTeK (Cognition‐Behaviour‐Technology) Research Lab, Université Côte d'azur, Nice France; ^2^ ki:elements GmbH, Saarbrücken Germany; ^3^ German Center for Neurodegenerative Diseases (DZNE) ‐ Rostock/Greifswald, Rostock Germany; ^4^ ki elements UG, Saarbrücken Germany; ^5^ Department of Psychiatry and Psychotherapy, Technical University of Munich, Munich Germany; ^6^ University of Edinburgh and UK DRI, Edinburgh United Kingdom; ^7^ German Center for Neurodegenerative Diseases (DZNE), Berlin Germany; ^8^ Department of Psychiatry and Psychotherapy, Charité, Charitéplatz 1, Berlin Germany; ^9^ Department of Neurodegenerative Diseases and Geriatric Psychiatry, University of Bonn Medical Center, Bonn Germany; ^10^ German Center for Neurodegenerative Diseases (DZNE), Bonn, Venusberg‐Campus 1, 53127 Bonn, Germany, Bonn Germany; ^11^ Department for Neurodegenerative Diseases and Geriatric Psychiatry, University Hospital Bonn, Bonn Germany; ^12^ German Centre for Neurodegenerative Diseases (DZNE), Bonn Germany; ^13^ University Hospital Carl Gustav Carus, Technische Universität Dresden, Dresden Germany; ^14^ German Center for Neurodegenerative Diseases (DZNE), Dresden Germany; ^15^ German Center for Neurodegenerative Diseases (DZNE), Goettingen Germany; ^16^ Department of Psychiatry and Psychotherapy, University Medical Center, University of Goettingen, Goettingen Germany; ^17^ Neurosciences and Signaling Group, Institute of Biomedicine (iBiMED), Department of Medical Sciences, University of Aveiro, Aveiro Portugal; ^18^ University Medical Center, Georg August University, Goettingen Germany; ^19^ Department of Psychiatry, Medical Faculty, University of Cologne, Cologne Germany; ^20^ German Center for Neurodegenerative Diseases (DZNE), Bonn Germany; ^21^ Excellence Cluster on Cellular Stress Responses in Aging‐Associated Diseases (CECAD) University of Cologne, Cologne Germany; ^22^ University Hospital Bonn (UKB), Clinic for Neurology, Bonn Germany; ^23^ German Center for Neurodegenerative Diseases (DZNE), Magdeburg Germany; ^24^ Institute of Cognitive Neuroscience, University College London (UCL), London United Kingdom; ^25^ Institute of Cognitive Neurology and Dementia Research (IKND), Otto‐von‐Guericke University, Magdeburg Germany; ^26^ Department of Neurology, University of Bonn, Bonn Germany; ^27^ Department of Neurodegeneration and Geriatric Psychiatry, University Hospital Bonn, Bonn Germany; ^28^ German Center for Neurodegenerative Diseases (DZNE), Tuebingen Germany; ^29^ Section for Dementia Research, Hertie Institute for Clinical Brain Research and Department of Psychiatry and Psychotherapy, University of Tuebingen, Tuebingen Germany; ^30^ Université Côte d’Azur, Cognition Behaviour Technology Lab (CoBTeK), Nice France; ^31^ University Medical Center Rostock, Rostock Germany; ^32^ Deutsches Zentrum für Neurodegenerative Erkrankungen e. V. (DZNE) Rostock/Greifswald, Rostock Germany; ^33^ CoBTek (Cognition‐Behaviour‐Technology), Université Côte d'Azur, Nice France

## Abstract

**Background:**

Speech and language impairments are associated with cognitive decline in neurodegenerative dementias, particularly Alzheimer’s Disease (AD), where subtle speech changes may precede clinical dementia onset. As clinical trials prioritize early identification for disease‐modifying treatments, digital biomarkers for timely screening become imperative. Digital speech‐based biomarkers can be employed for screening populations at the earliest AD stages. An automated phone‐based screening battery has been created, encompassing speech‐related neurocognitive tests (Semantic Verbal Fluency and Verbal Learning Test). This allows the extraction of speech‐based biomarkers alongside classical cognitive scores. This study aims to validate digital speech biomarkers in early‐stage AD by comparing them to traditional evaluation methods.

**Method:**

Within the PROSPECT‐AD project, speech and gold‐standard clinical data were obtained from the German DELCODE and DESCRIBE cohorts. We used data from N = 14 healthy controls (HC), N = 75 participants presenting with Subjective Cognitive Decline (SCD) and N = 18 participants presenting with amnestic Mild Cognitive Impairment (aMCI). Spearman rank correlations were computed between speech biomarkers and gold‐standard clinical measures. Kruskal‐Wallis test assessed group differences and regression analysis adjusted for age, sex and education, associated domain‐specific speech biomarkers and cognitive assessment scores.

**Result:**

There was a significant difference in the speech biomarker for cognition composite score (ki:e SB‐C) between diagnostic groups (x2(2) = 18.06, p <0.001). Significant correlations were found between ki:e SB‐C and all global anchor scores including MMSE (r = 0.48, d = 0.97, p <0.001), CDR‐SoB (r = ‐0.49, d = ‐0.98, p <0.001) and PACC5 (r = 0.56, d = 1.12, p <0.001) (Figure 1). All domain‐specific biomarker composite scores (memory, executive function, processing speed) significantly correlated with CDR, with strongest correlations found with the memory biomarker. All correlations remained significant when controlling for age, sex and education. Finally, based on the regression analysis results, domain‐specific biomarkers were significantly associated with respective domain‐specific anchors (Clock Drawing, TMT‐A/B, Digits Span, Figure Drawing, ADAS‐Cog, Verbal Fluency) (Table 1).

**Conclusion:**

Findings support prior research, emphasizing speech biomarkers as a promising tool for remote early‐stage AD screening, with potential implications for scalable screening in research trials and healthcare.